# Spatial navigation, episodic memory, episodic future thinking, and theory of mind in children with autism spectrum disorder: evidence for impairments in mental simulation?

**DOI:** 10.3389/fpsyg.2014.01411

**Published:** 2014-12-05

**Authors:** Sophie E. Lind, Dermot M. Bowler, Jacob Raber

**Affiliations:** ^1^Autism Research Team, Department of Psychology, Durham UniversityDurham, UK; ^2^Autism Research Group, Department of Psychology, City University LondonLondon, UK; ^3^Departments of Behavioral Neuroscience, Neurology, and Radiation Medicine, Division of Neuroscience, Oregon National Primate Research Center, Oregon Health & Science UniversityPortland, OR, USA

**Keywords:** autism spectrum disorder, episodic future thinking, episodic memory, mental simulation, scene construction, self-projection, spatial navigation, theory of mind

## Abstract

This study explored spatial navigation alongside several other cognitive abilities that are thought to share common underlying neurocognitive mechanisms (e.g., the capacity for self-projection, scene construction, or mental simulation), and which we hypothesized may be impaired in autism spectrum disorder (ASD). Twenty intellectually high-functioning children with ASD (with a mean age of ~8 years) were compared to 20 sex, age, IQ, and language ability matched typically developing children on a series of tasks to assess spatial navigation, episodic memory, episodic future thinking (also known as episodic foresight or prospection), theory of mind (ToM), relational memory, and central coherence. This is the first study to explore these abilities concurrently within the same sample. Spatial navigation was assessed using the “memory island” task, which involves finding objects within a realistic, computer simulated, three-dimensional environment. Episodic memory and episodic future thinking were assessed using a past and future event description task. ToM was assessed using the “animations” task, in which children were asked to describe the interactions between two animated triangles. Relational memory was assessed using a recognition task involving memory for items (line drawings), patterned backgrounds, or combinations of items and backgrounds. Central coherence was assessed by exploring differences in performance across segmented and unsegmented versions of block design. Children with ASD were found to show impairments in spatial navigation, episodic memory, episodic future thinking, and central coherence, but not ToM or relational memory. Among children with ASD, spatial navigation was found to be significantly negatively related to the number of repetitive behaviors. In other words, children who showed more repetitive behaviors showed poorer spatial navigation. The theoretical and practical implications of the results are discussed.

## Introduction

It has been proposed that a common, core network of brain regions (within the medial temporal lobe, precuneus, posterior cingulate cortex, retrosplenial cortex, temporal-parietal junction, lateral prefrontal cortex, and occipital cortex) underlies several high-level cognitive abilities including (a) certain types of spatial navigation, (b) remembering past events (episodic memory), (c) imagining future events (episodic future thinking/foresight/prospection), and (d) theory of mind (ToM) (e.g., Buckner and Carroll, 2007; Hassabis and Maguire, 2007; Spreng et al., 2009). At the cognitive level, Buckner and Carroll suggest that each of these functions involve “self-projection”—the ability to mentally simulate alternative perspectives. Alternatively, Hassabis and Maguire argue that the common underlying process (with the exception of ToM) is “scene construction”—the ability to generate and maintain a complex and coherent scene or event through binding the various (possibly multimodal) elements of a scene into a coherent whole (cf. Baddeley, 2000, 2002). The notions of self-projection and scene construction, which originate primarily from the field of neuroscience, closely echo the longer-standing notion of “mental simulation.” From this perspective, Shanton and Goldman (2010) have suggested that whereas ToM involves *inter*-personal simulation, episodic remembering and episodic future thinking involve *intra*-personal simulation (also see Schacter et al., 2008), and others have highlighted the importance of mental simulation in spatial navigation (e.g., Thorndyke and Hayes-Roth, 1982; Chersi et al., 2013).

Spatial navigation—or the ability to find one's way around an environment—can be supported by external representations, such as physical maps, or by internal, mental representations, generated from memory (Wolbers and Hegarty, 2010). The latter is known as “memory-guided” navigation. Researchers have identified several memory-guided navigation strategies, including “route-based” and “survey-based” strategies (Thorndyke and Hayes-Roth, 1982). Route-based navigation relies on incrementally learned, inflexible, egocentric (person-centered) representations of specific sequences of landmarks, junctions, left/right turns etc. On the other hand, survey-based navigation relies on flexible, allocentric (world-centered) representations, or “cognitive maps” (Tolman, 1948), of the spatial layout of the environment. Survey-based navigation is the specific form of navigation that is hypothesized to involve scene construction/self-projection/mental simulation.

The scene construction and self-projection theories rely heavily on support from neuroimaging studies of typically developing adults that suggest common brain regions are implicated during spatial navigation, episodic memory, episodic future thinking, and ToM tasks (e.g., Spreng et al., 2009). In terms of cognitive developmental studies of children, as far as we are aware, none have explored all four of these abilities in the same sample, and none appear to have explored relations between spatial navigation and any of the other three abilities. However, there is some evidence for developmental associations between episodic memory and episodic future thinking (e.g., Quon and Atance, 2010), and episodic memory and ToM (e.g., Perner et al., 2007), but not episodic future thinking and ToM (Hanson et al., 2014). If each of these abilities does rely on a common underlying cognitive process, their development would necessarily be constrained by development in that underlying process. In that case, one might predict that each ability should develop in parallel, at a similar rate. (Consistent with this analysis, episodic memory, episodic future thinking, and ToM all appear to undergo some significant developments at around the age of 4 years). On the other hand, this may not turn out to be the case, given that they also each involve their own unique cognitive processes (e.g., successful spatial navigation may also involve *planning* a route, in addition to generating a cognitive map of the environment), which may develop at different rates.

The scene construction, self-projection, and mental simulation theories are interesting and important theoretical proposals in their own right, but they may also be relevant to our understanding of autism spectrum disorder (ASD), a condition characterized by impairments in social communication and behavioral flexibility (American Psychiatric Association, 2013). People with ASD are often considered to have difficulties with “imagination” (e.g., Ten Eycke and Müller, 2014) but this is a rather broad, and often ill-defined concept (variously encompassing the notions of creativity, generativity, visual imagery, and/or the capacity for pretense). More specifically, we have hypothesized that ASD involves core impairments in scene construction and self-projection, which may underpin (or at least contribute to) a range of impairments in high-level cognitive processes that seem to involve some form of mental simulation (Lind et al., 2013, 2014). Along similar lines, Oberman and Ramachandran (2007) have suggested that individuals with ASD may have difficulties with mirror-neuron-based social simulation (i.e., *inter*-personal simulation, in Shanton and Goldman's, 2010, terms). However, this proposal is narrower in scope than our own, and only relates to ToM—not spatial navigation, episodic memory, or episodic future thinking.

The hypothesis that ASD involves core impairments in scene construction/self-projection/mental simulation is yet to be fully empirically tested, but several existing research findings are consistent with it. Previous studies of spatial navigation in ASD have reported mixed results and have suffered from problems such as inadequate ASD/comparison group matching on performance IQ (Prior and Hoffman, 1990; Fornasari et al., 2013) or sex (Edgin and Pennington, 2005), or incomplete reporting of results (Caron et al., 2004), making interpretation of findings inconclusive (see Lind et al., 2013, for a detailed evaluation). However, in a recent study, we found that intellectually high-functioning adults with ASD were moderately and significantly impaired in spatial navigation, relative to sex, age, and IQ matched neurotypical, comparison adults (Lind et al., 2013). Participants completed the “memory island” spatial navigation task (Rizk-Jackson et al., 2006; Piper et al., 2010), which involved asking them to find their way around a computer-simulated, island environment using a joystick, and to locate a series of target objects on the island. During an initial training (or “visible”) phase, the locations of the object were indicated by large, easily visible flags. This phase provided an opportunity to construct a cognitive map of the environment, to learn the locations of the target objects, and to learn the task. This phase did not require memory-guided navigation and could be completed using a “locomotor guidance” strategy—an online process that allows one to travel to a visible beacon (such as a flag) kept in constant view (Foo et al., 2005). Hence, performance during this phase indexed participants' ability to manage non-central task demands, such as comprehension of task instructions and proficiency with the joystick. This phase was immediately followed by the test (or “hidden”) phase in which participants had to locate the target objects without the aid of the flag markers. Here, participants had to rely solely on their cognitive map of the environment and their memory for the location of the target object (see Lind et al., 2013 for further explanation). It was found that participants with ASD had no difficulty locating the objects during the “visible” phase of the task, but had considerable difficulty with doing so during the “hidden” phase, as reflected by a tendency to search for the objects in the wrong areas of the island and to take longer, less efficient, routes. This suggests a specific difficulty with generating a cognitive map from a ground-based perspective.

Recently, Maras et al. (2014) explored the ability of intellectually high-functioning adults with ASD to recreate and scan a previously seen physical map of an island in their mental imagery. Performance of participants with ASD was very similar to that of closely matched comparison participants, suggesting that they preserved the spatial properties of the map in their mental images. This suggests that people with ASD are able to *hold* cognitive maps *in mind*, if the topographical layout of those maps is initially provided for them in a concrete visual form. Thus, difficulties with spatial navigation, as manifested in the Lind et al. (2013) study, appear to originate in actually *generating* cognitive maps themselves from their own ground-based memories of the layout of an environment. Such an interpretation is consistent with the idea that scene construction/self-projection/simulation is impaired among individuals with the disorder.

In addition to difficulties with navigation, we have also found that adults with ASD have impaired episodic memory, episodic future thinking, ability to imagine fictitious scenes, and ToM (Lind and Bowler, 2010; Lind et al., 2014; but see Crane et al., 2013). This research (as well as other research that has demonstrated impaired episodic memory and ToM in adults with ASD: e.g., Happé, 1994; Bowler et al., 2000) is consistent with the idea that self-projection/scene construction/mental simulation is impaired in adults with ASD.

However, less is known about these abilities in *children* with the disorder (with the exception of ToM, which has been explored in countless studies and is consistently found to be delayed in children with ASD; e.g., Yirmiya et al., 1998). As explained above, studies of spatial navigation in children with ASD do not provide clear evidence either for or against the idea that this ability is impaired. The limited existing evidence on episodic memory suggests that this does appear to be impaired in children with ASD (e.g., Lind and Bowler, 2009). Only one previous study has explored both episodic memory and episodic future thinking among *children* with ASD. Jackson and Atance (2008) and Terrett et al. (2013) conducted a pilot study of episodic future thinking but not episodic memory in children with ASD, but inadequate ASD/comparison group matching makes it difficult to interpret the results definitively. Terrett et al. asked children with ASD as well as sex-, age-, and IQ-matched typically developing children to complete the Adapted Autobiographical Memory Interview. This involved asking them to respond to cue words (e.g., “friend” or “naughty”) by providing verbal descriptions of past events that they had previously experienced and future events they were likely to experience. Consistent with the proposal that episodic memory and episodic future thinking are impaired in ASD, children with ASD were indeed found to produce fewer details overall. But, given the particular type of coding used in the study, as the authors themselves acknowledge, these between group differences could potentially have been explained by differences in narrative skills, which were not assessed as part of the study. Moreover, because Terrett et al. did not verify response accuracy, it is difficulty to be sure whether the participants' descriptions were based on accurate memory for the past or likely accurate episodic future thinking of the future.

Thus, although there is some preliminary evidence, it is currently unclear whether the difficulties with navigation, episodic memory, episodic future thinking, and ToM observed in adults with ASD are the later emergent outcome of widespread, interdependent atypical cognitive development and behavior, or whether such difficulties originate and are reliably evident in childhood. If already present in childhood, this may imply that difficulties with scene construction/self-projection/mental simulation are causally significant in the ASD cognitive profile (see Lind et al., 2013, for further discussion). For this reason, it is important to explore these abilities in children.

We are not aware of any research that has explored spatial navigation, episodic memory, episodic future thinking, and ToM in the same sample of children with ASD (or indeed any typically developing sample). The current study aimed to establish whether these abilities are concurrently impaired in ASD, and whether they are interrelated. If our hypothesis is correct and ASD involves core impairments in the capacity for scene construction/self-projection/mental simulation, we would expect to see significant impairments in spatial navigation, episodic memory, episodic future thinking, and ToM among children with ASD. We may also see (a) negative relations between each of these abilities and quantitative measures of ASD features, and (b) positive relations among spatial navigation, episodic memory, episodic future thinking, and ToM.

For the current study, children with and without ASD were asked to complete a battery of experimental tasks, including the memory island spatial navigation task, which we have previously employed with adults with ASD (Lind et al., 2013), as explained above; an event description task designed to assess episodic memory and episodic future thinking (based on Quon and Atance, 2010); and a version of the ToM animations task (Abell et al., 2000). With respect to the memory island task, given that only the “hidden” phase of the task requires genuine memory-guided navigation, involving the generation of a cognitive map (and, we hypothesize, scene construction/self-projection/mental stimulation), we expected to see ASD-specific difficulties in the hidden condition specifically.

The event description task was modeled on a task developed by Quon and Atance (2010). It involved asking participants to provide short verbal descriptions of particular events. Across three conditions, we assessed (a) autobiographical episodic memory, (b) autobiographical episodic future thinking, and (c) autobiographical semantic event knowledge. We predicted that children with ASD would perform less well across all three conditions but particularly so with respect to episodic memory and episodic future thinking. We did not predict intact performance in the semantic event knowledge condition (even though this should not involve scene construction/self-projection/mental stimulation) because previous research has shown autobiographical semantic memory and event schema knowledge to be impaired in children with ASD (Bruck et al., 2007; Loth et al., 2008), a deficit which may be related to broader impairments in self-awareness (Lind, 2010).

In order to assess ToM, we opted to use a version of the widely used (see White et al., 2011, for a review) “animations” task (Abell et al., 2000) because unlike many standard measures of ToM (e.g., false belief tasks), it is sensitive to ToM impairments among intellectually high-functioning individuals with ASD (e.g., Williams et al., 2013), and variation in ToM skills among typically developing children. The task requires participants to describe interactions between two triangles, as portrayed in a series of silent, animated video clips. We employed both a “metalizing” condition, comprising clips that were intended to evoke descriptions involving the attribution of mental states, such as belief, intention, and deception, as well as a “goal-directed” condition, comprising clips that were intended to evoke descriptions involving the attribution of physical agency and intentional action. Although both conditions are thought to involve ToM to some extent, the metalizing condition is thought to involve “higher-level” aspects of ToM. On the basis of abundant previous research and the hypothesis that self-projection or mental simulation is required for ToM (self-projection/mental simulation should allow one to “put oneself in the triangles' shoes”—i.e., take their perspective—to attribute mental states to them), we predicted that children with ASD would perform less well than typically developing children in both conditions.

In addition to the main experimental tasks referred to above, we also included measures of “central coherence” and “relational memory.” It is well established that people with ASD frequently show a detail-focused perceptual/cognitive style—termed “weak central coherence” by some researchers (e.g., Happé and Frith, 2006)—which can make it difficult for them to “see the forest for the trees.” It seems plausible that weak central coherence could be related to difficulties with scene construction in ASD (given that scene construction involves generating mental representations that involve binding together multiple, rich details which form a coherent scene), and may therefore be related to concomitant difficulties with spatial navigation, episodic memory, and episodic future thinking. Following Shah and Frith (1993), we obtained a measure of central coherence by asking participants to complete two versions of the block design subtest of the Wechsler intelligence scales. In this test, participants must recreate 2D geometric patterns using 3D colored blocks. Shah and Frith argued that people with ASD tend to perceive these patterns in a piecemeal fashion; hence the constituent parts are obvious to them. By contrast, typically developing individuals tend to see the whole design and do not automatically see the constituent parts. These researchers reasoned that by separating out the target patterns into their component parts—corresponding to individual blocks—the performance of typical individuals should be improved. On the other hand, people with ASD should not benefit from this manipulation because they already see the design in a piecemeal way. Indeed, this is what Shah and Frith found (and interpreted as evidence of weak central coherence in ASD). Thus, in the current study we asked participants to complete segmented and unsegmented versions of block design and obtained a difference score to provide an index of central coherence. Our expectation was that typically developing children should show greater central coherence (i.e., the segmentation manipulation should improve their performance more) than children with ASD.

We were also interested in exploring relational memory or memory “binding” —i.e., the ability to encode in and retrieve from memory the *associations between* features (Chalfonte and Johnson, 1996)—given that previous research has suggested this may be impaired in adults with ASD (Bowler et al., 2014), and that it is likely to be a pre-requisite for scene construction, given that it is multi-featural by nature. Relational memory was assessed using a recognition memory task (based on Lloyd et al., 2009) in which the to-be-remembered items were isolated items (line drawings), isolated (patterned) backgrounds, or combinations of items and backgrounds. We predicted that children with ASD would show significantly diminished recognition memory for *combinations* of items and backgrounds (which relies on relational memory) but intact memory for individual items (given that previous research indicates that item recognition is generally intact in ASD; see Boucher et al., 2012, for a review).

Previously, we have speculated that difficulties with episodic future thinking (Lind and Bowler, 2010) and navigation (Lind et al., 2013) may be connected with the behavioral inflexibility that characterizes ASD. For example, we questioned whether difficulties with flexibly imagining possible future situations could contribute to over-reliance on rigid routines, and whether difficulties with survey-based spatial navigation might result in overreliance on inflexible, route-based navigation and insistence on following well learned routes among some individuals. Alternatively, behavioral inflexibility might actually play a causal role in the atypical development of episodic future thinking and or/navigation. Either way, it is important to establish whether there is any empirical relation between these variables as we aimed to do in the current study.

## Materials and methods

### Participants

Twenty intellectually high-functioning children with ASD and 20 typically developing comparison children participated, after they and their parents had given written, informed consent to take part. Ethical approval for this study was obtained from Durham University Psychology Research Ethics Committee. Participants received gift vouchers (worth £15) to thank them for their time. Participants with ASD were recruited via (a) the Database of Children with ASD Living in the North East of England, (b) local primary schools, (c) an advert in an email newsletter to staff at Durham University, and (d) word of mouth. Comparison participants were recruited through local primary schools.

Inclusion criteria included having a full-scale IQ = 80, being aged 6–12 years, and having no neurological or psychiatric disorders, other than ASD. Participants in the ASD group had all received formal diagnoses of autistic disorder (*n* = 15) or Asperger's disorder (*n* = 5), according to DSM-IV criteria (American Psychiatric Association, 2000). All documented diagnostic information was checked thoroughly by the researchers and provided sufficient information to ensure diagnostic criteria were met in each case.

Although it is becoming increasingly common to use the Autism Diagnostic Observation Schedule-Generic (ADOS-G; Lord et al., 2000) and the Autism Diagnostic Interview-Revised (ADI-R; Lord et al., 1994) to “confirm” ASD diagnoses for research purposes, we decided not use these instruments in the present study for several reasons. Firstly, they have surprisingly low specificity (i.e., rate of detecting true negatives). In the largest study of its kind (*N* = 1039, compared to *N* = 381 and *N* = 50 in the initial ADOS-G and ADI-R validation studies, respectively), Risi et al. (2006) found that although the sensitivity (i.e., rate of detecting true positives) of the ASD criteria on the ADOS-G and the autism criteria on the ADI-R (the ADI-R does not have separate ASD criteria) was reasonably high across several independent samples (ADOS-G: 72.4–96.9; ADI-R: 82.7–95.1%), the specificity was sometimes extremely low (ADOS-G: 10.0–77.9%; ADI-R: 40.9–72.8%). Hence, these instruments classify large numbers of non-spectrum individuals (who have other disorders) as autistic. In our view, this is unacceptably low specificity to allow diagnostic confirmation for our own research purposes. Arguably, for the purpose of cognitive-experimental research (such as the present study), specificity is of critical importance because we need to be confident that our ASD sample comprises genuine cases. Secondly, there is often poor agreement between the instruments (e.g., Bishop and Norbury, 2002; Ventola et al., 2006). Hence, using ADOS-G ASD criteria and/or ADI-R autism criteria for selecting research participants is problematic. We take the view that expert clinical judgment (i.e., formal diagnosis) is a far more valid and reliable criterion for inclusion in research such as this.

We agree with Bishop (2011); Bishop (May 30) that the ADOS-G and ADI-R are “not optimal for diagnosing ASD for research purposes,” and that “assessments such as the...Social Responsiveness Scale, which treat autistic features as dimensions rather than all-or-none symptoms, seem better suited to this task than the existing gold standards.” Hence, to assess current ASD features among children with ASD and the presence of ASD-like features among comparison children for the current study, participants' parents/carers were asked to complete the Social Responsiveness Scale, Second Edition (SRS-2, Constantino and Gruber, 2012). Scores on this 65-item questionnaire provide a valid and reliable quantitative indicator of ASD features. The SRS-2 (using a raw score cut-off of ≥ 60) has high sensitivity (93%) *and* specificity (91%) (Constantino and Gruber, 2012), and (unlike the ADI-R) is independent of IQ (Constantino et al., 2003). In addition to the psychometric advantages of the SRS over the ADOS-G and ADI-R, it also takes a fraction of the time (~15 min, as opposed to ~1 and 3 h, respectively) to administer, thereby minimizing the test burden on participants. Only two participants with ASD missed the ASD cut-off (each scoring 57 points). Data analyses were conducted including and excluding these participants (groups remained matched on baseline variables) and results were not substantively altered. Thus, all 20 participants with ASD were retained in the final sample. All comparison participants scored below the ASD cut-off. Thus, none showed any sign, according to parents or carers, of manifesting significant ASD-like traits. To obtain a thorough index of behavioral inflexibility, participants' parents were also asked to complete the 43-item Repetitive Behavior Scale—Revised (RBS-R) questionnaire (Bodfish et al., 2000).

Using the Wechsler Abbreviated Scale of Intelligence (WASI; Wechsler, 1999), the groups were matched closely for verbal and non-verbal IQ. Using the Test for Reception of Grammar, Second Edition (TROG-2, Bishop, 2003), the groups were matched for structural language ability. The groups were also matched for sex, χ^2^_(1, *N* = 40)_ = 0.14, *p* = 0.71, ϕ = 0.06, and chronological age. Importantly, all effect sizes (*r* values) associated with group differences in baseline characteristics of sex, age, IQ, and linguistic ability were negligible to small in magnitude, indicating close matching. Participant characteristics and matching statistics are presented in Table 1.

**Table 1:** **Participant characteristics (means, standard deviations, and inferential statistics for between-group differences)**.

**Characteristic**	**ASD (*n* = 20)**	**Comparison (*n* = 20)**	***t***	***p***	***r***
Sex (male: female)	16: 4	15: 5			
Age (years)	8.67 (1.37)	8.32 (0.91)	0.94	0.353	0.15
VIQ	104.05 (13.54)	107.15 (5.29)	0.95	0.350	0.15
PIQ	105.35 (18.05)	109.60 (14.22)	0.83	0.431	0.13
FSIQ	105.65 (16.34)	109.05 (8.68)	0.82	0.418	0.13
TROG-2^a^	90.40 (13.23)	93.50 (8.21)	0.89	0.380	0.13
SRS-2^b^	110.65 (33.67)	22.00 (12.57)	11.03	<0.001	0.88
RBS-R^c^	34.30 (21.54)	1.40 (2.01)	6.80	<0.001	0.73

*Note. VIQ, verbal IQ; PIQ, performance IQ; FSIQ, full scale IQ; TROG-2, Test for Reception of Grammar – Version 2; SRS-2, Social Responsiveness Scale – Second Edition; RBS-R, Repetitive Behavior Scale – Revised*.

a Standard Score;

b Raw score;

c *Overall score*.

### Overview of procedures

Participants completed the task battery over two sessions in the following order: WASI (including the unsegmented version of block design), TROG-2, relational memory task, spatial navigation task, event description task, ToM task, and segmented block design.

### Memory island spatial navigation task

#### Materials and procedures

**Figure 1:**
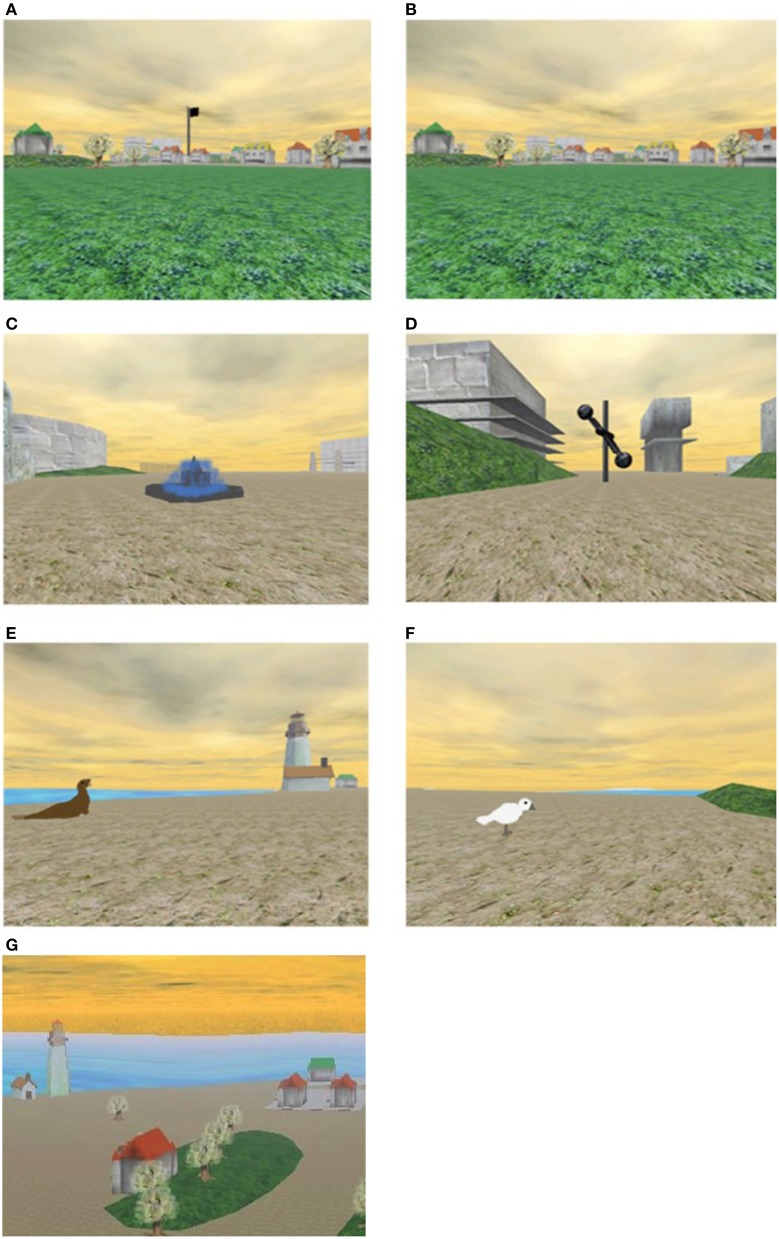
**Screen shots from the Memory Island spatial navigation task. (A,B)** Show the same view as it appeared in the visible and hidden conditions of the experiment, respectively; **(C–F)** show the target objects used during the visible trials; **(D)** shows the target object used for the hidden trials. **(G)** Shows an aerial view of part of the island (this is purely for illustrative purposes—participants never saw the island from this perspective). Part of this figure is reproduced from Lind et al. (2013), published by American Psychological Association and reprinted with permission.

Participants were asked to navigate within a computer-simulated, three-dimensional, island environment, measuring 347 × 287 meters (see Figure 1 for screenshots).

The task was presented on a laptop computer (Sony Vaio; 16.4 inch screen) and included not only a visual (full color) depiction of the surroundings, including buildings and other landmarks, but also accompanying “nature” sounds, such as birdsong and moving water. Participants sat at a comfortable distance from the screen (~50 cm).

Participants could explore the virtual environment using a Microsoft Sidewinder joystick, which allowed them to determine their direction and speed of travel. At the outset of the task, participants were presented with the following on-screen instructions, which were also read aloud by the experimenter:

You will cruise on a virtual island. In each trial, you will start in the same position, but you may be looking in a different direction. Your mission is to find a mysterious object hidden somewhere on the island. To do that you need to look closely at what's on the island. Try to make a map in your head of the island and where the mysterious objects are located on the island. If you cannot find the mysterious object within 2 min, an arrow will help guide you to it. Once you have found it, you must stand next to it and wait for the game to end.

Participants were then asked if they had any questions, to ensure they fully understood the requirements of the task.

#### Visible condition

During the first phase of the task, participants completed four “visible” trials. In this condition, target items were marked by large flags, which could be clearly seen from a considerable distance. Across visible trials 1, 2, 3, and 4, the target objects were a sculpture, seal, seagull, and fountain, respectively. Each target was assigned a unique location. On a given trial, only one target was visible. At the outset of each trial, participants were instructed to locate the flag and move toward it to find the target object. If they were unable to locate it within 2 min, an arrow appeared to direct them to it. Once the object was found, the participant was required to stand next to it, and the word “Remember” appeared on the screen, prompting them to try to memorize its location.

#### Hidden condition

The visible phase was immediately followed by the hidden phase, comprising four hidden trials. In this condition, no flag markers were present, and participants were required to find their way to the same target object (sculpture) on each trial (the location was identical to the visible trial). The repeated use of the same target across hidden trials provided participants with continued opportunity for learning (protecting against floor effects). The target object was indicated to the participant, in the form of an on-screen visual image, at the outset of each trial. As for visible trials, if a participant was unable to locate the target object within 2 min, an arrow appeared to direct them to it.

For both visible and hidden trials, the starting location was always the center of the island. The starting orientation was varied across trials but these variations remained constant across participants. In other words, participants started each trial facing a different direction—a feature of the task, designed to eliminate reliance on inflexible (egocentric), procedurally memorized sequences of turns, or stimulus–response associations, rather than (allocentric) topographical knowledge of the layout of an environment. Target locations were kept constant for all participants. The time taken to complete the task varied according to how quickly participants completed each trial (10–15 min for most participants). The experimenter remained present throughout.

#### Dependent measures

Participants' movements were recorded in time-stamped, coordinate files. Several dependent variables were calculated for each trial, including (a) proportion of time spent within the target quadrant; (b) latency to reach the target (seconds); (c) proportion of successful trials in which the target was located within the 2 min trial time; (d) velocity (virtual units/second); (e) path length (virtual units); and (f) cumulative distance to the target (virtual units).

For purposes of analysis, the island was divided into four equal quadrants (rather like a pie chart divided into quarters) as is commonly done for target quadrant analysis in water maze-type navigation tasks. If a participant has successfully learned the location of the target object, he/she typically will spend more time searching in the correct quadrant. Hence, a higher *proportion of time spent in the target quadrant* indicates better performance and a more efficient route. *Latency* to reach the target simply provides a measure of how long it takes a participant to reach the target object. *Proportion of successful trials* provides the most basic indicator of task performance. Successful trials were defined as trials in which the target was located within 2 min (i.e., before the arrow appeared on screen to direct the participant to the target object). *Velocity* is not itself a measure of task success, but several other dependent variables, depend to some extent on velocity. These variables include proportion of successful trials (it is more difficult to reach the target within 2 min if one is traveling very slowly), and latency (if one is traveling quickly, one should be able to reach the target object in a shorter time). By contrast, proportion of time spent in the target quadrant is independent of velocity. In general, a shorter *path length* indicates more efficient navigation—if an individual knows where they are going they can travel directly there. However, it is important to note that, although this measure is independent of velocity, path length is not necessarily the optimal performance measure because shorter paths do not *necessarily* indicate task success (i.e., locating the target within 2 min). The final measure, *cumulative distance to target*, is obtained by sampling the distances from the position of the participant on the island to the target object, 10 times per second for the duration of the trial, and summing these values. It provides an indication of proximity to the target during navigation (and hence navigational search efficiency). Hence, lower values indicate better performance.

Data were not analyzed on a trial-by trial basis because starting-orientation (which varied across trials) influences the level of difficulty. Thus, for the purpose of data analysis, performance was collapsed across the four visible, and four hidden trials, respectively. Average scores, rather than total scores, for each condition were used throughout (to aid comparisons with previous studies).

### Event description (episodic memory/episodic future thinking) task

#### Design and materials

The event description task was modeled on a task developed by Quon and Atance (2010). It involved asking participants to provide short, verbal descriptions of particular events. Across three conditions—past events, future events, and semantic knowledge conditions—three types of test question phrasing (e.g., what did you do…/what are you going to do…/what do you do…) were used to probe (a) episodic memory of specific past events, (b) episodic simulation of specific future events, and (c) semantic, script-based knowledge of events of a given type, respectively. We were careful to select a mixture of frequently occurring and relatively infrequently occurring events for which the accuracy or likelihood of a child's response could be easily judged by their parents. The events used included breakfast, evening meal, bedtime, food shopping, going to the park, having a meal in a restaurant, Christmas, own birthday, and school trip.

The task was fully counterbalanced across participants to ensure no systematic bias in terms of (a) which events appeared in which of the three conditions (e.g., we ensured the event “breakfast” was used for the past condition for some children, the future condition for some children, and the semantic condition for some children), and (b) the order of presentation of events/conditions. See Supplementary Material for the phrasing used for each event type in each condition.

#### Procedure

***Experimental task***. The experimenter and child sat opposite one another in a quiet room in their school or in a laboratory at Durham University. The experimenter began the task by saying, *“I'm very curious about all the different things you do! So I would like to ask you some questions about some of the things you do and some of the places you go to. Okay, let's start!”* Prior to asking about each event, the experimenter would say, for example, *“Let's talk about what you eat for breakfast”* or *“I want to ask you about bedtime.”* She then used the event scenario questions as specified for the specific versions of the task being used for that child.

If the participant responded with *“I don't know”* or did not respond at all, the experimenter used the following prompt (but only once): *“Let's think really hard about…”* and then repeated the original question. If the child still failed to respond, the experimenter proceeded to next question. If the participant gave a very general response such as *“play,”* the experimenter prompted for a more specific response (e.g., *“What did you/do you/are you going to play?”*). If the child failed to elaborate, the experimenter moved onto the next question. If the participant gave a specific response, the experimenter gave the one-time prompt, *“Anything else?”* All responses were recorded on a solid-state audio recording device.

***Accuracy check***. Once the child had completed the task, the accuracy of their responses was checked with their parents as soon as possible. These checks were either done in person (for those children tested in a university laboratory) or by telephone or email (for children tested at school). For past and semantic condition questions, the experimenter provided the child's response and asked the parent if it was *accurate*. For future condition questions, she asked if it was *likely accurate*. More specifically, they were asked to provide responses on a 10-point scale (0 = did not happen/not likely to happen/completely accurate, 5 = uncertain, and 10 = definitely happened/will definitely happen/completely accurate).

For events that do not (by definition) occur on daily or annual basis (food shopping, park, restaurant, school trip), the experimenter asked the parent about how frequently the child experiences the event, to ensure there were not any systematic differences between the groups in this respect.

#### Scoring and reliability

All recordings of verbal responses were transcribed by the experimenter. These transcriptions were then coded by the experimenter and a second, blind coder on three dimensions: (1) whether the child gave a specific (i.e., indicative of episodic foresight/memory) response (response specificity); (2) whether the child's response was accurate/likely, according to parent report (response accuracy); and (3) the proportion of “script indicators” (linguistic markers indicative of semantic knowledge) in the response (proportion of script indicators). Extraneous aspects of children's responses that did not focus on the question were not coded.

***Response specificity***. If the child (a) failed to provide a response (e.g., “I don't know”), (b) gave an irrelevant or inappropriate response (e.g., “football” for breakfast event), or gave only a very broad, generic response (e.g., “everything” or “play” for birthday event) after the initial question and follow-up prompt, they were given a score of 0. If the child gave a specific response (e.g., “pizza” for evening meal event), they were assigned a score of 1. An overall response specificity proportion score for each condition was calculated based on the child's average score across the three questions in each condition. Inter-rater reliability for response specificity was high, Cohen's κ = 0.96.

***Response accuracy***. Scores of 0 were assigned to (a) responses that scored 0 on response specificity and (b) responses that parents judged to be inaccurate. Scores of 1 were assigned to responses that parents judged to be accurate/likely accurate. For the purpose of data analysis, ratings of 0–4 were deemed “inaccurate” (scoring 0), ratings of 6–10 were deemed “accurate” (scoring 1), and ratings of 5 (i.e., the parent was “uncertain”) were excluded and treated as missing data. An overall response accuracy proportion score for each condition was calculated based on the child's average score across the three questions in each condition.

***Proportion of script indicators***. The first step in calculating the proportion of script indicators was to identify the number of (a) script indicators (e.g., generalized present tense verbs; words such as “usually” and “sometimes”), (b) future temporal indicators (e.g., future tense verbs; words such as “tomorrow” and “next time”), and (c) past temporal indicators (e.g., past tense verbs; words such as “yesterday” and “last time”). See Quon and Atance (2010) for further detail. An overall script indicator proportion score for each condition was calculated based on the average number of script indicators children used across each question, relative to the total number of script, future temporal and past temporal indicators they used in that question. Inter-rater reliability for script indicator proportion score was high, Cronbach's α = 0.97.

### Theory of mind “animations” task

**Figure 2:**
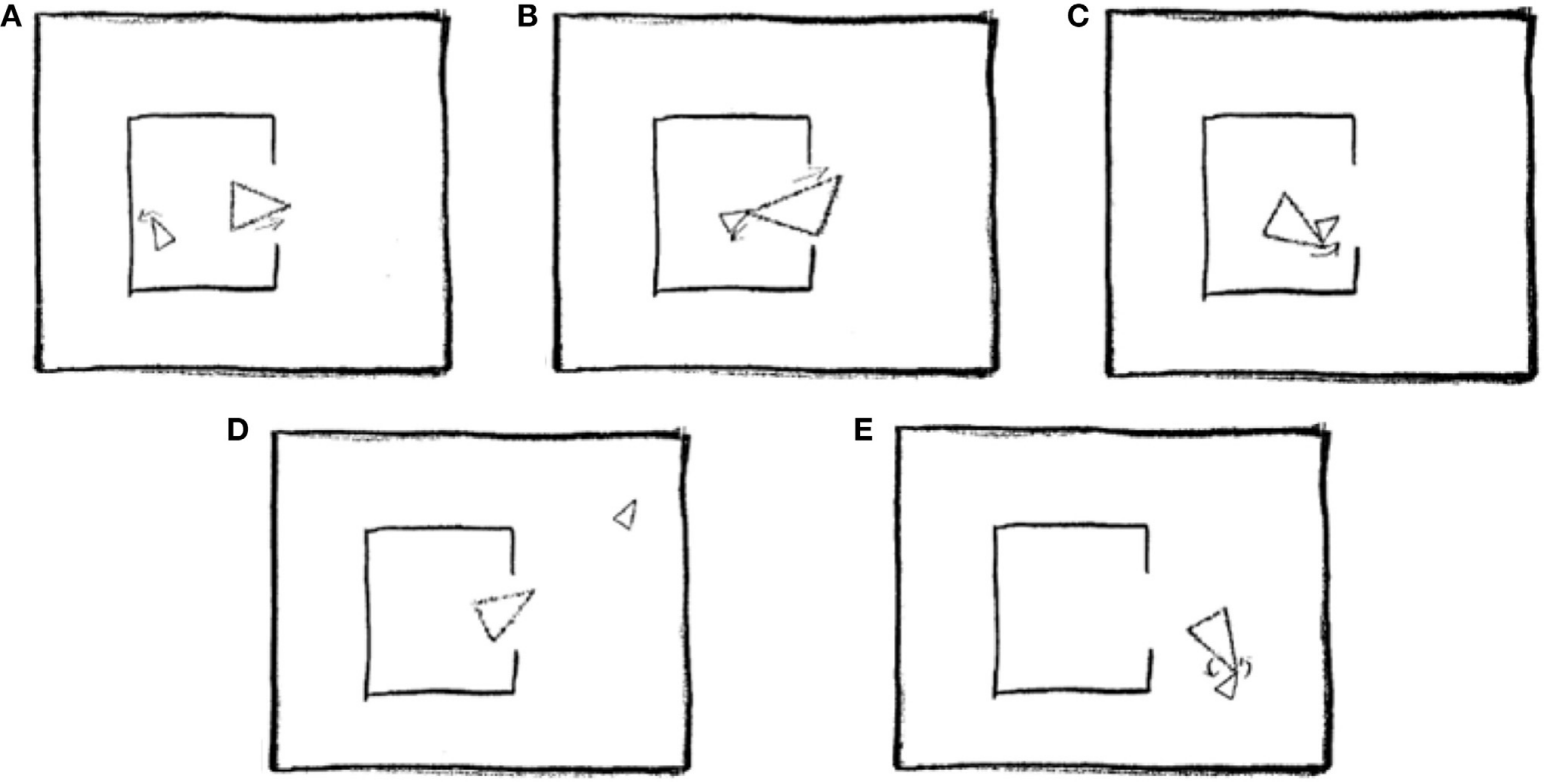
**Five stills taken from one of the animations scripted as Coaxing (mother and child): (A) Mother tries to interest child in going outside. (B)** Child is reluctant to go out. **(C)** Mother gently nudges child toward door. **(D)** Child explores outside. **(E)** Mother and child play happily together. Reprinted from Abell et al. (2000), Copyright Elsevier Science Inc., with permission from Elsevier.

As explained in the introduction, ToM was assessed using a short version of the “animations” task (Abell et al., 2000), which requires participants to describe interactions between two triangles, as portrayed in a series of silent video clips (see Figure 2 for an illustration).

For the metalizing condition, we employed the “coaxing” and “surprising” clips, and for the goal-direct condition, we employed the “following” and “fighting” clips. According to Abell et al.'s criteria, descriptions were assigned scores of 0, 1, or 2 according to their level of accuracy. Therefore, within each condition, the maximum score was 4 points. Inter-rater reliability for animations scores was high, Cronbach's α = 0.96.

### Relational memory task

#### Overview

The relational memory task involved study and yes/no recognition test phases, which were separated by a filler task (ensuring that short-term memory could not contribute to experimental task performance). Stimuli were simple line drawings of objects (“items”) and patterned backgrounds (“backgrounds”). During the study phase, children viewed three types of stimuli: (1) isolated items; (2) isolated backgrounds; and (3) combinations of items and backgrounds. During the test phase, they were presented with these same stimuli along with lures/distractors, which also included these three different types of stimuli. Isolated item and isolated background lures (seen at test only) were completely “new” (i.e., not seen at study) but combination lures were only partly “new” (in that the component items and backgrounds had appeared at study, but not in that combination). This ensured that participants could not achieve correct responses on combination stimuli by relying on memory for the item or background alone—they had to recognize the unique combination (relying specifically on relational binding).

#### Materials

In the first instance, a master set of 40 Snodgrass and Vanderwart (1980) line drawings (“items”) and 40 Microsoft PowerPoint “backgrounds” was collated. We selected items that we anticipated would be visually appealing to children (e.g., rabbit, kite, carrot). Only relatively simple patterns (e.g., red fabric, crinkly brown paper, water droplets) were used as backgrounds.

We then created five stimulus subsets, each containing eight items and eight backgrounds, randomly selected from the master set. Using a balanced Latin square, these subsets were used to create 10 counterbalanced versions of the task. Across these versions, each subset was rotated across the following trial types: (1) “isolated trials A” and (2) “isolated trials B”—here, items and backgrounds were presented individually at study and at test; (3) “combination trials”—here, items and backgrounds were presented as part of unique combinations that remained unchanged across study and test; (4) “combination + combination lure trials”—here, items and backgrounds were presented as part of combinations, but they were presented in one combination at study and a different combination at test; (5) “isolated lure trials”—here, items and backgrounds were presented individually at test only (they were not shown at study).

**Figure 3:**
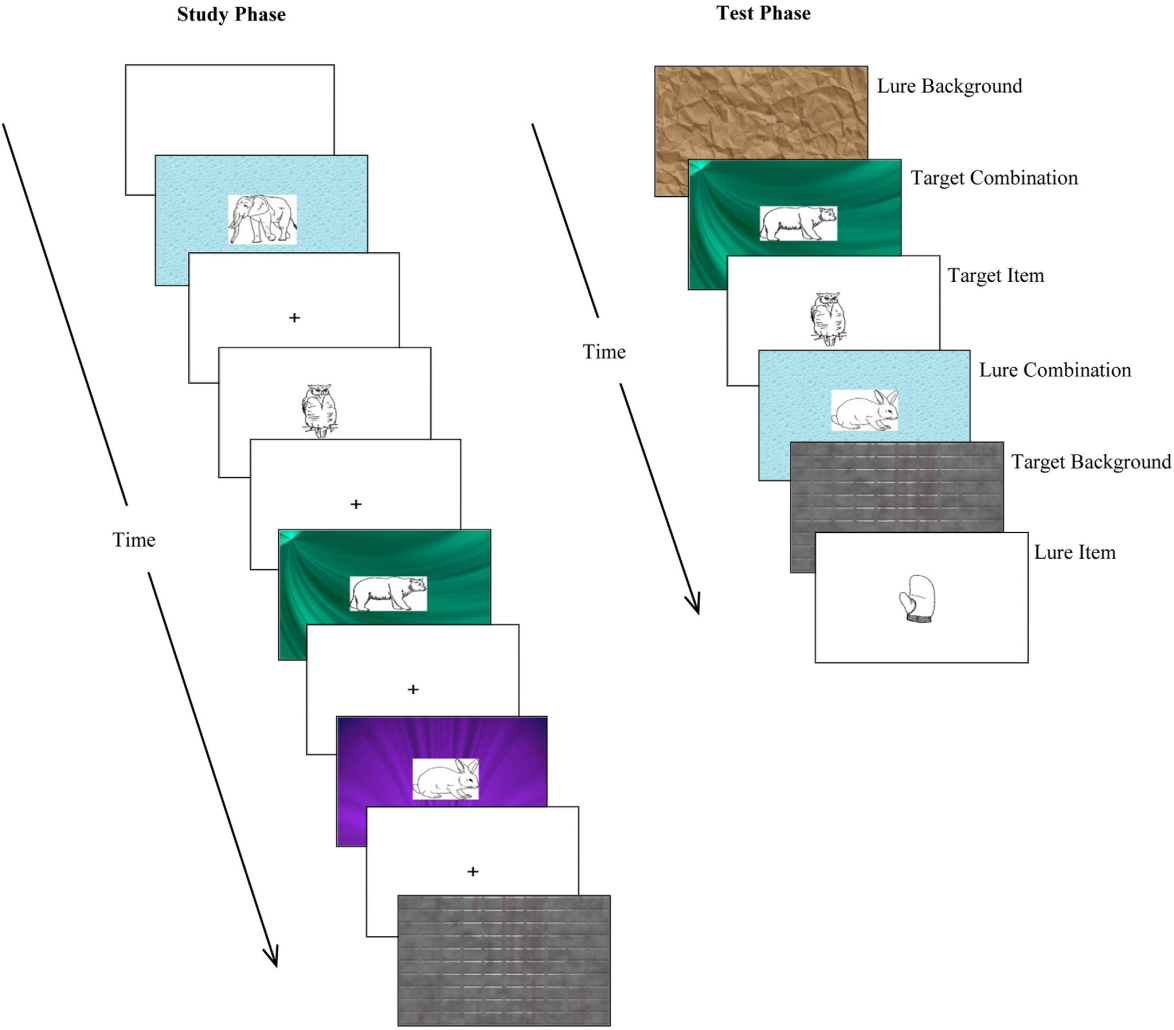
**Examples of stimuli presented during the relational memory task**. At study, participants were presented with three type of stimuli: isolated items (simple line drawings); isolated backgrounds (patterned backgrounds); and combinations of items superimposed on backgrounds. At test, participants were presented with previously studied stimuli (targets) as well as distractor stimuli (lures), and asked to make old/new recognition judgments.

For each version of the task, the stimulus subsets designated as “isolated A,” “isolated B,” “combination,” and “combination + lure” were presented in a pseudorandom order during the study phase (subject to the constraint that no more than three of each trial type—isolated item, isolated background, combination—appeared sequentially). During the test phase, “isolated A,” “isolated B,” “combination,” “combination + combination lure” (with new combinations), and “isolated lure” trials were, again, presented in a pseudorandom order (subject to the constraint that no more than three of each trial type appeared in a row). Thus, 48 and 64 stimulus slides were presented during the study and test phase, respectively. Examples of the stimuli presented during the study and test phases are shown in Figure 3.

All experimental task stimuli were presented on a laptop computer using Microsoft PowerPoint software.

#### Procedure

***Training phase***. Before commencing the experimental task itself, participants completed a two-step training task to ensure they fully understood the task requirements. The materials used for training were completely different from those used for the experiment itself. At step 1, the experimenter explained that *“I'm going to show you some pictures of things and some backgrounds, and then I want to test your memory for them.”* At this point, she showed them two isolated items and two isolated backgrounds sequentially. Then she said, *“Now I'd like to see how many of those pictures and backgrounds you can remember”* and showed them the “old,” previously presented stimulus slides, along with one “new” (lure), isolated item and one “new,” isolated background one after another. For each slide, she asked “*Did you see that picture/background just now?”* If the child did not score 100% on step 1 of the training task, the experimenter provided feedback and tested them again with a second set of training materials.

At step 2 of the training task, the experimenter explained that “Now I'm going to show you some different pictures on different backgrounds and see whether you can remember which pictures belong on which backgrounds” and showed the child three item-background combinations sequentially, in each case saying, “See, that's where the [item] belongs.” Then she showed them one of the original item-background combinations and two new combinations, based on the remaining two old combinations, one after another. For each one, she asked, “Did you see that picture on that background just now? Is that where the [item] belongs?” As for step 1, if a child failed to score 100% on step 2, the experimenter provided feedback, and tested them again with a second set of training materials. Any child who could not achieve 100% on step 1 or 2 of the training phase would have been excluded from the experiment but no child fell into this category.

***Study phase***. Having successfully completed the training phase, participants progressed onto the experimental task. At this point, the experimenter explained that *“Now I'm going to show you lots more pictures and backgrounds and later on I'm going to see how many of them you can remember. Sometimes I'll show you pictures on their own, sometimes backgrounds on their own, and sometimes I'll show you pictures on backgrounds. For those ones, it's important to try to remember which background the picture belonged on.”* Each child was assigned to one of the 10 counterbalanced versions of the task and viewed the corresponding set of study phase stimulus slides. Each of the slides appeared on screen for 4 s. During each 1-s inter-stimulus interval, a plain white slide with a fixation-cross appeared.

***Filler task***. The filler task was completed immediately after the study phase. The materials consisted of 15 slides presented on Microsoft PowerPoint, each containing two large circles. The circles were colored red, green, or blue and the child's task was to say on which side (left/right) the green circle appeared on each slide. Including time taken to explain the filler task and the time taken to explain the test phase of the experimental task, the interval between study and test was 3–4 min.

***Yes/no recognition test phase***. After the filler task, the experimenter said, *“Now I'd like to see how many pictures and backgrounds you can remember from earlier”* and showed the participant the test phase stimulus slides and lure slides, in each case asking, *“Did you see that picture/background earlier?”* or “*Did you see that picture on that background just now? Is that where the [item] belongs?”* when appropriate (isolated items, isolated backgrounds, and combinations were interspersed). The slides were again presented using PowerPoint but no timings were used. Once the child has responded “yes” or “no” to a particular slide, the experimenter moved onto the next slide manually (i.e., this phase was self-paced). Responses were recorded manually using a pen and scoring sheet.

***Dependent measures and scoring***. The key dependent measures on the task were, hit rates (proportions of studied items/backgrounds/combinations correctly identified as “old”), false alarm rates (proportion of lure items/backgrounds/combinations incorrectly identified as “old”), and corrected hit rates (hit rate minus false alarm rate) across the item, background, and combination conditions.

### Central coherence task—segmented vs. unsegmented block design

#### Materials and procedure

**Figure 4:**
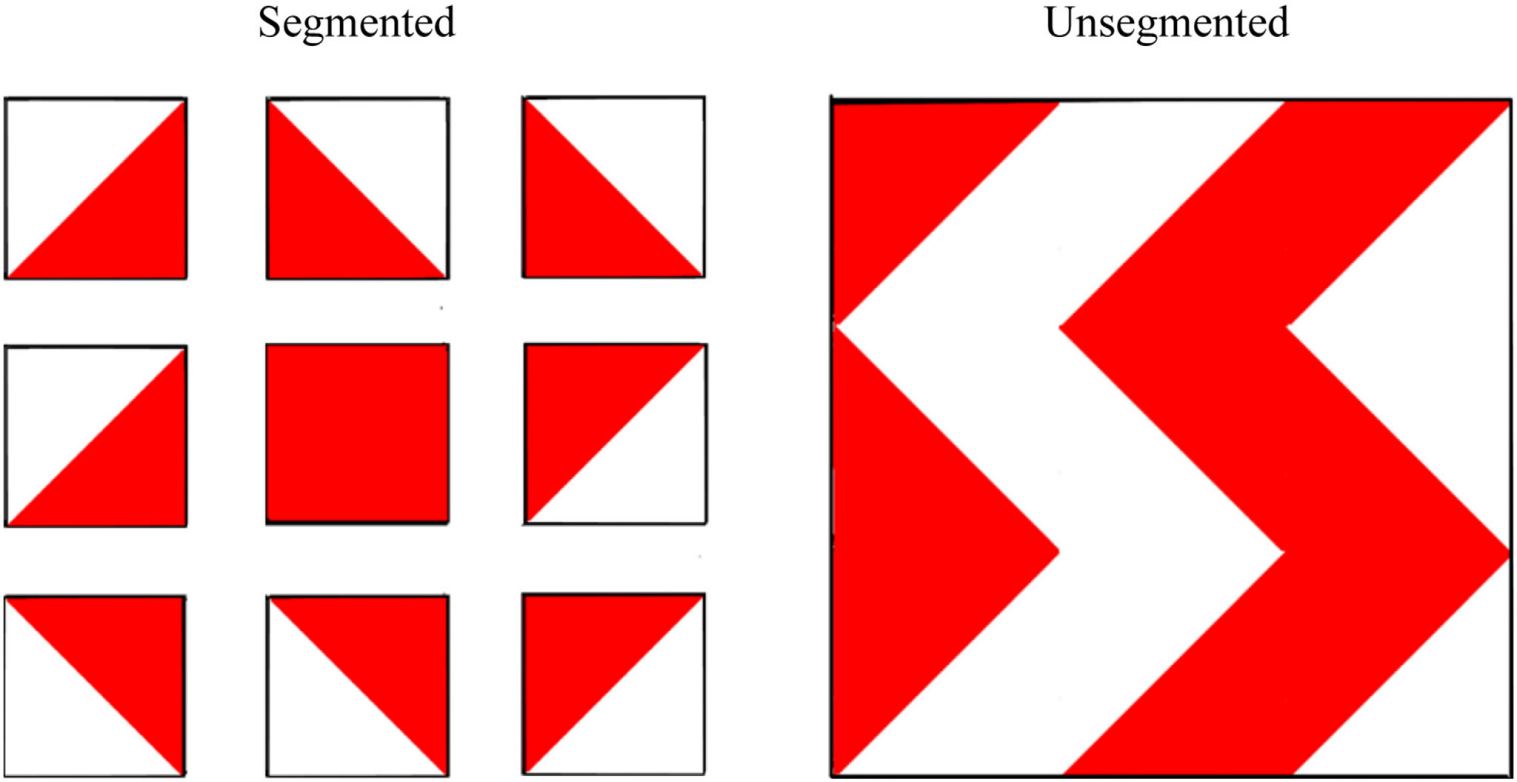
**Examples of segmented and (standard) unsegmented block design patterns used to assess central coherence**. Participants were asked to recreate the 2D patterns using 3D colored blocks, each with two red sides, two white sides, and two half red-half white sides. *Wechsler Abbreviated Scale of Intelligence*. Copyright © 1999 NCS Pearson, Inc. Reproduced with permission. All rights reserved. *“Wechsler Abbreviated Scale of Intelligence*” and “*WASI*” are trademarks, in the US and/or other countries, of Pearson Education, Inc. or its affiliates(s).

The unsegmented version of the block design task was administered and scored, according to standard instructions, as part of the WASI. To ensure consistency between the unsegmented and segmented versions, rather than using the stimulus booklet itself, the designs were reproduced exactly as they appear in the WASI stimulus booklet on separate pieces of paper measuring approximately 20 cm by 21 cm. Each 2D “block” was reproduced to appear 3.9 cm by 3.9 cm and each design was laminated. The block design patterns for the segmented version were reproduced precisely from the Wechsler Intelligence Scale for Children—Fourth UK Edition (WISC-IV UK; Wechsler, 2004). For this task, the designs were also reproduced on separate 20 cm by 21 cm pieces of laminated paper with 2D squares of 3.9 cm by 3.9 cm in size. This time, however, each 2D block was separated by 1.7 cm of white space, effectively segmenting the designs into their individual blocks. See Figure 4 for an illustration of the task.

#### Scoring

To obtain a metric of central coherence, difference scores were created by subtracting unsegmented raw scores from segmented raw scores. Thus, higher difference scores indicate stronger central coherence because they imply the participant benefited to a greater extent from the manipulation to the stimuli.

### Data analysis

A standard alpha level of 0.05 was used to determine statistical significance. All reported significance values are for two-tailed tests (except those associated with correlation analyses or *t*-tests, where directional hypotheses were made). We report *r* values as measures of effect size for continuous variables and Cramer's *V* for categorical variables (≥0.10 = small, ≥0.30 = moderate, ≥0.50 = large; Cohen, 1969).

## Results

### Spatial navigation task

A series of six 2 (Group: ASD, comparison) × 2 (Condition: visible, hidden) mixed-design ANOVAs were conducted to explore differences in (a) proportion of time in the target quadrant, (b) latency, (c) proportion of successful trials, (d) velocity, (e) path length, and (f) cumulative distance to target, respectively. The results of these analyses and full descriptive statistics are reported in Table 2.

**Table 2:** **Descriptive (means and standard deviations) and inferential (Group [ASD, comparison] × Condition [visible, hidden] mixed-design ANOVA) statistics for spatial navigation dependent measures**.

**Dependent measure**	**Condition**	**Descriptive statistics**			**Inferential statistics**								
					**Group**			**Condition**			**Group × condition**		
		**ASD (*n* = 19)**	**Comparison (*n* = 20)**	**Total**	***F*_(1, 37)_**	***p***	***r***	***F*_(1, 37)_**	***p***	***r***	***F*_(1, 37)_**	***p***	***r***
Proportion of time in target quadrant	Visible	0.91 (0.04)	0.89 (0.07)	0.90 (0.05)									
	Hidden	0.79 (0.17)	0.85 (0.08)	0.82 (0.13)									
	Total	0.85 (0.13)	0.87 (0.08)	0.86 (0.11)									
					0.61	0.439	0.13	16.16	**<0.001**	0.55	3.72	0.062	0.30
Latency to reach target (seconds)	Visible	66.12 (13.94)	60.24 (7.52)	63.10 (11.37)									
	Hidden	87.33 (26.49)	68.15 (10.83)	77.50 (22.03)									
	Total	76.73 (23.49)	64.20 (10.04)	70.30 (18.86)									
					7.61	**0.009**	0.41	33.62	**<0.001**	0.69	7.01	**0.012**	0.40
Proportion of successful trials	Visible	0.99 (0.06)	1.00 (0.00)	0.99 (0.04)									
	Hidden	0.86 (0.19)	0.96 (0.09)	0.91 (0.16)									
	Total	0.92 (0.15)	0.98 (0.06)	0.95 (0.12)									
					5.59	**0.023**	0.36	12.41	**0.001**	0.50	3.84	0.058	0.31
Velocity (virtual units/second)	Visible	7.07 (1.05)	7.51 (0.70)	7.30 (0.90)									
	Hidden	7.60 (1.00)	8.02 (0.68)	7.82 (0.87)									
	Total	7.33 (1.05)	7.77 (0.73)	7.56 (0.92)									
					2.80	0.103	0.27	27.17	**<0.001**	0.65	0.01	0.92	0.02
Path length (virtual units)	Visible	443.65 (23.32)	438.72 (34.51)	441.12 (29.31)									
	Hidden	634.33 (167.05)	537.28 (79.26)	584.56 (137.02)									
	Total	538.99 (152.23)	488.00 (78.31)	512.84 (122.07)									
					4.56	**0.039**	0.33	61.98	**<0.001**	0.79	6.29	**0.017**	0.38
Cumulative distance to target (virtual units)	Visible	30989 (7933)	28349 (4186)	29635 (6353)									
	Hidden	46373 (20316)	33307 (7673)	39672 (16392)									
	Total	38681 (17093)	30828 (6597)	34654 (13343)									
					7.16	**0.011**	0.40	19.61	**<0.001**	0.59	5.15	**0.029**	0.35

*Values in bold are significant at the p < 0.05 level*.

**Figure 5:**
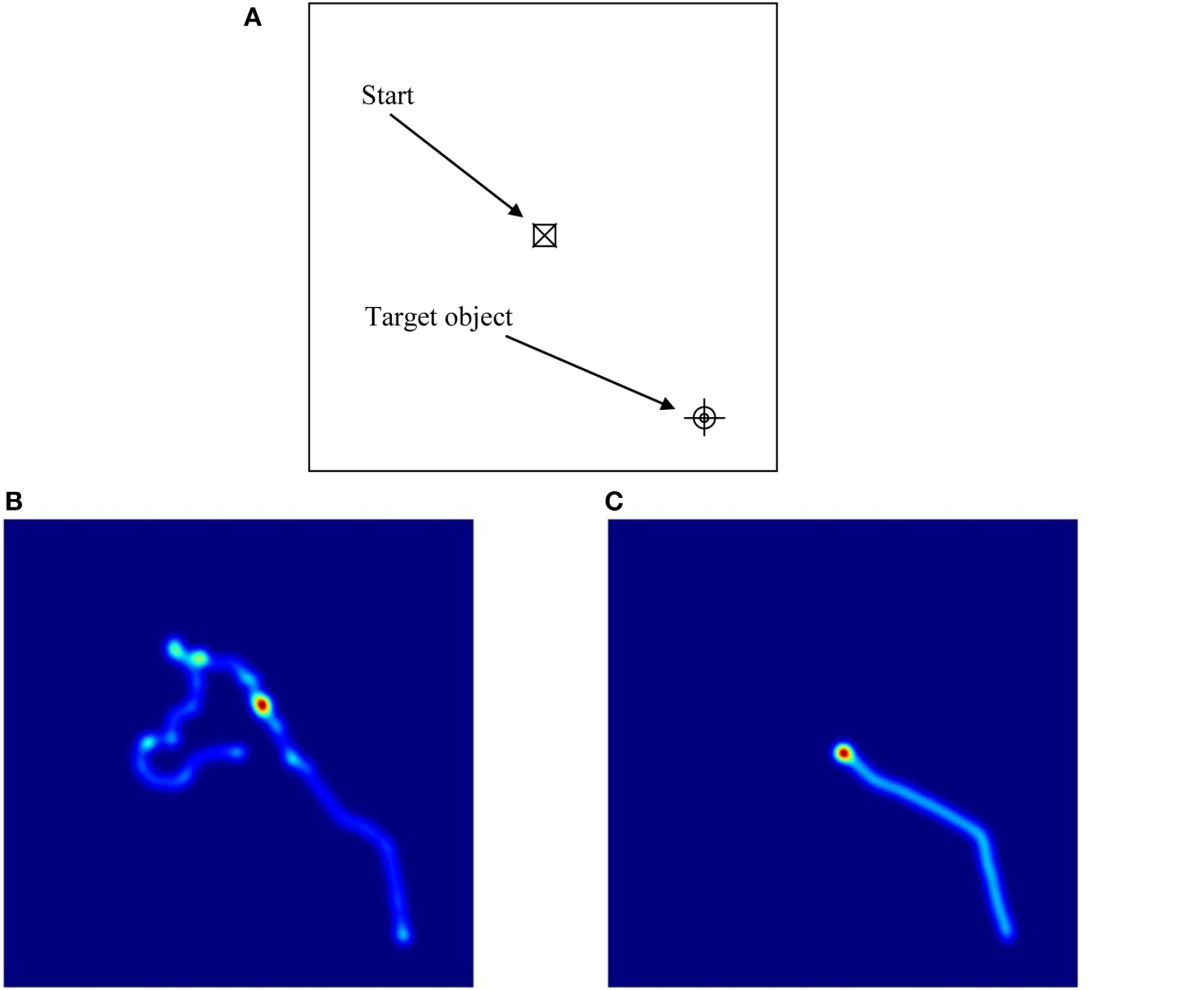
**Illustrations of performance on hidden trial 1 of the Memory Island navigation task**. As shown in **(A)**, participants started the trial in the center of the island (and center-point of the figure) and had to find their way to the target, which is positioned at the bottom right of the figure. **(B,C)** are heat maps illustrating the routes taken by the best **(C)** and worst **(B)** performing participants. In **(B)**, the participant starts off in the opposite direction to the target and has to double-back on himself to eventually find it. In **(C)**, the participant takes a moment to correctly orient himself and then travels directly to the target.

These ANOVAs revealed significant main effects of Group on latency, proportion of successful trials, path length, and cumulative distance (or proximity of search area) to target (each associated with moderate effect sizes), but not on proportion of time in the target quadrant, or velocity (each associated with small effect sizes). These results reflect the fact that the ASD group took longer overall to find the targets, successfully completed fewer trials, took longer routes, and covered a broader search area than the comparison group. There were also significant main effects of Condition on all of the five dependent variables (each associated with moderate to large effect sizes). These results reflect the fact that in the hidden trials, participants spent a smaller proportion of time in the target quadrant, took longer to find the targets, successfully completed fewer trials, traveled more quickly, took significantly longer routes, and covered a broader search area compared to the visible trials (see Figure 5 for heat maps of the best and worse performing participants in hidden trial 1).

Most relevant to our predictions, significant or marginally significant interaction effects between Group and Condition were found for all variables (each associated with moderate effect sizes), except for velocity (associated with a negligible effect size). Inspection of the descriptive statistics (see Table 2) suggests the significant or marginally significant interaction effect on proportion of time spent in the target quadrant was driven by the fact that the ASD group performed *better* than the comparison group in the visible condition (*r* = 0.17), but performed *less well* in the hidden condition (*r* = 0.22). The significant interaction effect on latency seems to be driven by the fact that the ASD group showed only slightly longer latencies than the comparison group in the visible condition (*r* = 0.25) but substantially longer latencies in the hidden condition (*r* = 0.43). The marginally significant interaction effect on proportion of successful trials is likely to be explained by the fact that both groups performed at, or close to, ceiling in the visible condition (*r* = 0.12), but the ASD group successfully completed substantially fewer trials than the comparison group in the hidden condition (*r* = 0.32). The significant interaction effect on path length appears to be due to the fact that the groups showed very similar path lengths in the visible condition (*r* = 0.08) but the ASD group took substantially longer paths than the comparison group in the hidden condition (*r* = 0.35). Finally, the significant interaction effect on cumulative distance to target appears to be explained by the fact that group differences were larger in the hidden condition (*r* = 0.39) than the visible condition (*r* = 0.20).

### Event description (episodic memory/episodic future thinking) task

Before analyzing the main experimental results from the event description task, preliminary analyses were carried out to ensure there were no systematic group differences in how frequently the children experienced the events they were asked about, according to parent report. The frequency data are reported in Table 3.

**Table 3:** **Frequency at which children in each of the groups experienced each event type according to parental report**.

**Event**	**Group**	**Frequency of experiencing**								
		**Almost daily**	**At least weekly**	**At least fortnightly**	**At least monthly**	**At least every 3 months**	**At least every 6 months**	**At least annually**	**Less than annually**	**Never**
Food shopping	ASD	0	8	5	5	0	1	0	0	1
	Comparison	0	7	4	6	0	0	0	1	0
Park	ASD	0	7	5	6	0	0	0	2	0
	Comparison	2	8	3	4	1	0	0	0	0
Restaurant	ASD	0	2	6	11	0	0	0	0	1
	Comparison	0	2	7	6	2	1	0	0	0
School trip	ASD	0	0	0	1	0	19	0	0	0
	Comparison	0	0	0	0	0	17	1	0	0

Chi-square analyses revealed no significant differences in how frequently children in each group experienced food shopping trips, trips to the park, meals at restaurants, or school trips, all χ^2^s_(5, *N* = 38)_ = 5.88, *p*s = 0.318, all *V*s = 0.39. Thus, any observed group differences in experimental task performance cannot be attributed to group differences in frequency of relevant experiences.

The three main dependent measures on the event description task were response specificity, response accuracy, and proportion of script indicators. A series of three 2 (Group: ASD, comparison) × 3 (Condition: past, future, semantic) mixed-design ANOVAs were conducted to explore differences in each of these variables. The results of these analyses and full descriptive statistics are reported in Table 4.

**Table 4:** **Descriptive (means and standard deviations) and inferential (Group [ASD, comparison] × Condition [past, future, script] mixed-design ANOVA) statistics for the event description (episodic memory/episodic future thinking) task**.

**Dependent measure**	**Condition**	**Descriptive statistics**			**Inferential statistics**											
					**Group**				**Condition**				**Group × condition**			
		**ASD**	**Comparison**	**Total**	***F***	***df***	***p***	***r***	***F***	***df***	***p***	***r***	***F***	***df***	***p***	***r***
Response specificity	Past	0.88 (0.20)	0.98 (0.07)	0.93 (0.15)												
	Future	0.87 (0.27)	0.93 (0.14)	0.90 (0.22)												
	Script	0.92 (0.21)	0.97 (0.10)	0.94 (0.17)												
	Total	0.89 (0.23)	0.96 (0.11)	0.93 (0.18)												
					3.06	1,38	0.089	0.27	0.86	2,76	0.429	0.11	0.29	2,76	0.753	0.06
Response accuracy	Past	0.88 (0.20)	0.98 (0.08)	0.93 (0.16)												
	Future	0.83 (0.28)	0.94 (0.13)	0.89 (0.22)												
	Script	0.90 (0.22)	0.98 (0.08)	0.94 (0.17)												
	Total	0.87 (0.23)	0.97 (0.10)	0.92 (0.19)												
					4.79	1,36	**0.035**	0.34	1.35	2,72	0.265	0.14	0.10	2,72	0.908	0.04
Proportion of script indicators	Past	0.08 (0.13)	0.07 (0.13)	0.07 (0.13)												
	Future	0.32 (0.23)	0.36 (0.18)	0.34 (0.21)												
	Script	0.66 (0.24)	0.61 (0.25)	0.64 (0.25)												
	Total	0.35 (0.32)	0.35 (0.29)	0.35 (0.30)												
					0.04	1,38	0.840	0.03	73.79	2,76	**<0.001**	0.70	0.52	2,72	0.597	0.08

*Values in bold are significant at the p < 0.05 level*.

These analyses revealed a marginally significantly main effect of group on response specificity (associated with a small effect size; reflecting greater specificity among the comparison group), a significant effect of group on response accuracy (associated with a moderate effect size; reflecting higher accuracy among the comparison group), and a non-significant effect of group on proportion of script indicators (associated with a negligible effect size). It is important to note that larger underlying group differences in response specificity and response accuracy may have been masked by the near-ceiling level performance of the comparison group.

Non-significant main effects of condition were observed for response specificity and response accuracy (associated with small effect sizes), but a clear effect of condition was observed for proportion of script indicators (associated with a large effect size). This reflects the very low proportion of script indicators used in the past condition, the higher proportion used in the future condition, and the very high proportion used in the semantic condition. No significant interaction effects between group and condition were observed for any of the three dependent variables (each associated with negligible effect sizes).

### Theory of mind (animations) task

Animations task performance was analyzed using a 2 (Group: ASD, comparison) × 2 (Condition: metalizing, goal-directed) mixed-design ANOVA. This revealed (contrary to expectations) a non-significant main effect of Group, *F*_(1, 38)_ = 0.11, *p* = 0.747, *r* = 0.05, and a non-significant interaction effect between Group and Condition, *F*_(1, 38)_ = 0.33, *p* = 0.570, *r* = 0.09, reflecting similar levels of performance among the groups in both the goal-directed (ASD: *M* = 3.00, *SD* = 0.79; comparison: *M* = 3.05, *SD* = 0.94) and metalizing conditions (ASD: *M* = 1.05, *SD* = 1.19; comparison: *M* = 0.85, *SD* = 1.04). There was, however, a strong and significant main effect of condition, *F*_(1, 38)_ = 90.71, *p* < 0.001, *r* = 0.84, reflecting better performance in the goal-directed than metalizing condition.

### Relational memory task

A series of three 2 (Group: ASD, comparison) × 3 (Condition: item, background, combination) mixed-design ANOVAs were conducted to explore differences in hit rate, false alarm rate, and corrected hit rate, respectively. The results of these analyses and full descriptive statistics are reported in Table 5.

**Table 5:** **Descriptive (means and standard deviations) and inferential (Group [ASD, comparison] × Condition [item, background, combination] mixed-design ANOVA) statistics for relational memory task dependent measures**.

**Dependent measure**	**Condition**	**Descriptive statistics**			**Inferential statistics**								
					**Group**			**Condition**			**Group × condition**		
		**ASD**	**Comparison**	**Total**	***F*_(1, 38)_**	***p***	***r***	***F*_(2, 76)_**	***p***	***r***	***F*_(2, 76)_**	***p***	***r***
Hit rate	Item	0.74 (0.21)	0.81 (0.17)	0.78 (0.19)									
	Background	0.68 (0.21)	0.76 (0.14)	0.72 (0.18)									
	Combination	0.57 (0.28)	0.67 (0.23)	0.62 (0.26)									
	Total	0.67 (0.24)	0.75 (0.19)	0.71 (0.22)									
					2.61	0.114	0.25	9.54	**<0.001**	0.33	0.08	0.923	0.10
False alarm rate	Item	0.10 (0.23)	0.04 (0.10)	0.07 (0.18)									
	Background	0.03 (0.07)	0.03 (0.07)	0.03 (0.07)									
	Combination	0.34 (0.21)	0.41 (0.25)	0.37 (0.23)									
	Total	0.15 (0.23)	0.16 (0.24)	0.16 (0.23)									
					0.05	0.822	0.04	54.21	**<0.001**	0.70	1.53	0.227	0.16
Corrected hit rate	Item	0.64 (0.28)	0.77 (0.20)	0.70 (0.25)									
	Background	0.66 (0.24)	0.73 (0.15)	0.69 (0.21)									
	Combination	0.23 (0.24)	0.26 (0.23)	0.25 (0.24)									
	Total	0.51 (0.32)	0.59 (0.30)	0.55 (0.31)	2.12	0.154	0.23	68.54	**<0.001**	0.69	0.61	0.547	0.09

*Values in bold are significant at the p < 0.05 level*.

Each of these analyses revealed non-significant main effects of Group (associated with negligible to small effect sizes), significant main effects of Condition (associated with moderate to large effect sizes), and non-significant interactions between Group and Condition (associated with negligible to small effect sizes). The significant main effects of Condition across all three dependent measures reflect the fact that, in line with expectations, recognition memory was substantially better in the isolated item and background conditions than in the combination condition.

### Segmented vs. unsegmented block design (central coherence) task

The mean (*SD*) unsegmented block design raw scores for the ASD and comparison groups were 24.22 (16.22) and 27.55 (12.34), respectively. The mean (*SD*) segmented scores were 43.50 (18.71) and 53.15 (7.91), respectively. Consistent with the theory of weak central coherence (Happé and Frith, 2006), participants with ASD obtained lower segmented/unsegmented block design difference scores (*M* = 19.28, *SD* = 12.40) than comparison participants (*M* = 25.60, *SD* = 11.26). In other words, they were less affected by the manipulation to the patterns than comparison participants (consistent with the idea that they already see the designs in terms of their component parts). An independent samples *t*-test indicated that this small difference was marginally statistically significant, *t*_(36)_ = 1.65, *p* = 0.054, *r* = 0.26.

### Relations between tasks

Finally, we analyzed the correlations among each of the main dependent variables and the measures of ASD features, within each group separately. For the purpose of these correlations, the following variables were used: SRS-2 raw score (measure of ASD features); RBS-R total score (measure of behavioral inflexibility); cumulative distance to target in the hidden condition (measure of memory guided spatial navigation); corrected hit rate in the combination condition (measure of relational memory); response accuracy in the past condition (measure of episodic memory); response accuracy in the future condition (measure of episodic future thinking); response accuracy in the semantic script knowledge condition (measure of semantic event knowledge); combined goal-directed and metalizing score (measure of ToM); segmented/unsegmented block design difference score (measure of central coherence). The results are displayed in a correlation matrix (see Table 6).

**Table 6:** **Correlations among the main dependent variables and measures of ASD features: *r*-values for the ASD group with *r*-values for comparison group shown in parentheses**.

	**Navigation**	**Relational memory**	**Episodic memory**	**Episodic future thinking**	**Semantic event knowledge**	**ToM**	**Central coherence^i^**
SRS-2 ^a^	−0.37 (0.34)	−0.17 (−0.16)	0.16 (0.19)	0.26 (−0.29)	0.35 (−0.39)	0.27 (0.06)	<0.01 (0.12)
RBS-R ^b^	−0.48^*^ (0.15)	0.10 (−0.39^*^)	0.28 (0.17)	0.07 (−0.20)	0.33 (0.17)	0.24 (−0.37)	0.07 (−0.12)
Navigation ^c^		−0.49^*^ (0.23)	0.07 (−0.17)	0.09 (−0.27)	0.10 (−0.06)	−0.12 (0.09)	−0.10 (0.06)
Relational memory ^d^			>0.01 (−0.11)	−0.02 (0.34)	−0.28 (−0.11)	−0.23 (0.35)	−0.19 (0.09)
Episodic memory ^e^				0.27 (−0.11)	0.53^*^ (−0.06)	0.15 (−0.18)	0.21 (0.26)
Episodic future thinking ^f^					0.48^*^ (0.11)	0.02 (0.26)	−0.31 (−0.38)
Semantic event knowledge^g^						0.13 (−0.02)	0.21 (−0.28)
ToM ^h^							−0.09 (−02)

*RBS-R, Repetitive behavior scale; SRS-2, Social Responsiveness Scale – Second Edition; ToM, theory of mind*.

* Significant at the p < 0.05 level;

a Raw score;

b Total score;

c Cumulative distance to target in the hidden condition;

d Corrected hit rate in the combination condition;

e Response accuracy in the past condition;

f Response accuracy in the future condition;

g Response accuracy in the semantic script knowledge condition;

h Combined goal-directed and metalizing score;

i *Segmented/unsegmented block design difference score*.

*Values in bold are significant at the p < 0.05 level*.

Notably, the predicted positive relations among spatial navigation, episodic memory, episodic future thinking, and ToM were not supported by the analyses, with non-significant results observed in each case. Within the ASD group, significant moderate-to-strong correlations were observed between (a) spatial navigation and behavioral inflexibility (negative relation; as predicted), (b) spatial navigation and relational memory (negative relation; contrary to predictions), (c) episodic memory and semantic event knowledge (positive relation; as expected), and (d) episodic future thinking and semantic event knowledge (positive relation; as expected). Within the comparison group, there was an unexpected significant, moderate, negative correlation between relational memory and behavioral inflexibility. None of the other correlations reached statistical significance.

## Discussion

Our first prediction that spatial navigation would be impaired among children with ASD was clearly supported by the data. Significant or marginally significant group (ASD/comparison) by condition (visible/hidden) interaction effects were observed for all of the dependent variables, apart from velocity, which is not itself regarded as a performance measure. This reflects the fact that children with ASD had difficulties with the task that were specific to the hidden condition, which unlike the visible condition, relied on memory guided navigation. The ASD group spent a smaller proportion of time in the target quadrant, took longer to find the targets, successfully completed fewer trials, took significantly longer routes, and covered a broader search area, compared to the typically developing group. This is only the second published study to directly demonstrate impairments in survey-based/cognitive-map-based navigation among individuals with ASD, relative to age, sex, and IQ matched typically developing individuals, and the first study to show it in *children* with the disorder (although impairments in other forms of spatial memory have been demonstrated in this population; Pellicano et al., 2011).

The current findings are broadly in line with our previous findings from a sample of intellectually high-functioning *adults* with ASD (Lind et al., 2013). Together, these studies suggest that spatial navigation impairments are relatively early to emerge in development and are long-standing, persisting into adulthood (of course, longitudinal research, and research with other paradigms would be needed to conclude this with greater certainty). However, it is notable that both the ASD and typically developing child groups employed in the current study performed notably better on the navigation task (which was identical) than did their adult counterparts (with similar IQs) employed in our previous study. Although we cannot be certain of the reason for this finding, one possibility is that children are more familiar with and proficient in navigating around computer-simulated environments because they have more experience with computer games. Overall though, these findings are consistent with our core hypothesis that self-projection/scene construction/mental simulation is diminished in ASD.

We also predicted that children with ASD would show impaired episodic memory and episodic future thinking in the event description task. There was a marginally significant difference between the groups in terms of response specificity but, more importantly, the ASD group showed a significant disadvantage on the key dependent measure, response accuracy, regardless of condition. Thus, children with ASD not only had more difficulty generating specific responses but also appeared to be confabulating past experiences and imagining implausible future experiences to a greater extent than typically developing children. This is an important and striking finding since few studies of memory, and none of episodic future thinking, in ASD have explored accuracy—the majority have assessed the level of detail (e.g., Lind et al., 2014) or response specificity (e.g., Crane and Goddard, 2008; Lind and Bowler, 2010) of verbal reports of events.

A notable exception is a study of autobiographical memory by Bruck et al. (2007), in which children with and without ASD were asked to recall life events (with accuracy of reports being established through parental report). They found that children with ASD produced significantly fewer event recall utterances that were confirmed by their parents, indicating reduced levels of episodic remembering. However, in general, children with ASD did not produce significantly more utterances that were “unconfirmed” by or “inconsistent” with parental report. This led Bruck et al. to conclude that children with ASD were no more likely than children without ASD to produce confabulated memories. However, in each past event narrative, children with ASD produced fewer utterances *per se* than children without ASD. Now, it is arguable that, for the purposes of judging the extent to which children with ASD confabulate, the most informative analysis would have been to explore the *proportion* of unconfirmed/inconsistent responses produced by participants *within* each group. A larger proportion of such responses by children with ASD would indicate a greater tendency to confabulate. To resolve this issue, we explored Bruck et al.'s (2007) participant responses to “specific” questions (which made up the majority of their interview) about each life event (see their Tables 2–5). Collapsing their data across age groups, we calculated the proportion of utterances made by participants from each diagnostic group that were inconsistent or unconfirmed. According to our calculations, across all four event-narratives, the proportion of such utterances was 0.53 (*SD* = 0.52) for the ASD group and 0.27 (*SD* = 0.46) for the comparison group. In this respect, the difference between the groups was statistically significant, with a moderate effect size, *t*_(64)_ = 2.71, *p* = 0.03, *d* = 0.54. Contrary to Bruck et al.'s (2007) conclusions, therefore, a substantial proportion of utterances made by participants with ASD about their previous life events could not be confirmed, suggesting significant confabulation. The results of the current study are consistent with this reanalysis of Bruck et al.'s data.

As explained in the introduction, Terrett et al. (2013) explored episodic memory and episodic future thinking among children with ASD. Their method was similar to the current method but the coding of responses was quite different. Terrett et al. coded responses according to *number of details* generated. Hence, we cannot establish whether their participants' verbal responses were based on accurate memory for the past or likely accurate episodic future thinking of the future. Only the current study has explored this. Nevertheless, together these studies suggest that episodic memory and episodic future thinking are impaired in children with ASD. The fact that impairments in these abilities have been observed in both children and adults, across different studies, suggests that, like navigation impairments, these difficulties are both early emerging and persistent through development.

The results did not support our prediction that ToM would be impaired among children with ASD—in fact, participants with ASD performed slightly better than typically developing children in the metalizing condition of the animations task. This was an unexpected but, by no means, unprecedented finding (e.g., Salter et al., 2008). This result may be at least partly explained by the fact that *both* groups found the task quite challenging. For instance, the mean accuracy score for the typically developing comparison group on the metalizing condition was just 21%. This is far lower than the mean obtained in Abell et al.'s (2000) original typically developing sample, who scored approximately 59%, despite the fact that participants from each study were of a similar mean age and ability level (Abell et al.'s study: mean CA = 8.5 years; mean FSIQ = 97; current study: mean CA = 8.32; mean FSIQ = 109). Thus, a trend toward floor effects in the metalizing condition may potentially have masked latent group differences in ToM in the current study. One notable difference between the current study and Abell et al.'s study is that we used two rather than four clips for each condition. Although this could potentially explain the discrepant findings, Abell et al.'s item analysis suggested that all clips within each condition were of an equivalent level of difficulty, so it seems unlikely that we had inadvertently selected the more difficult clips for the current study. Regardless of the reason for the current pattern of results, it is clear that these ToM findings do not provide any positive evidence for the suggestion that self-projection or inter-personal mental simulation are impaired in ASD. This stands in contrast to the majority of published research on ToM in ASD, which shows reliable impairments (and is in keeping with the hypothesis of impairments in self-projection and/or mental stimulation).

The results also failed to support our prediction of impaired relational memory in ASD—children with ASD not only showed recognition memory for *individual* items or backgrounds that was equivalent to sex, age, and IQ matched typically developing children (confirming previous research; e.g., Lind and Bowler, 2009; see Boucher et al., 2012, for a review), but also equivalent recognition memory for *combinations* of items and backgrounds. This suggests that, contrary to predictions; relational memory for pairs of elements is intact in children with ASD. This finding is unlikely to be due to lack of statistical power, given that our sample size exceeded that needed to obtain the recommended level of statistical power required to detect a genuine group difference if present (0.80; Cohen, 1992). Moreover, the effect sizes associated with group differences in recognition memory measures in the combination condition were all small in magnitude (and had high associated *p*-values). The current findings stand in contrast to those of Bowler et al. (2014), who observed a significant diminution in relational memory among adults with ASD. It is worth considering the possibility that although relational memory problems may not affect *children* with ASD, they may emerge as a developmental consequence of having ASD (see Karmiloff-Smith, 1997). However, arguably, a more plausible explanation for the discrepancies in results across these studies lies in the specific task demands of the experimental procedures employed in each of these studies, respectively. It is notable that the current results are consistent with the “task support hypothesis” (e.g., Bowler et al., 2004), which suggests that memory performance in ASD is facilitated when external “support” is provided at retrieval. Hence, (supported) tests of recognition memory or cued recall are considerably easier than (unsupported) tests of free recall for individuals with ASD. The current results show that children with ASD are able to *encode* the relations between pairs of features, and *retrieve* those relations in a highly supported recognition memory test. However, it remains a possibility that those successfully encoded relations may not be so readily retrieved if less support were provided at test (e.g., memory was tested using a free recall procedure). But even if this is true, this cannot be the complete explanation for the current null findings, since Bowler et al. also used a (supported) recognition memory procedure. It is also possible that although binding of simple binary relations in ASD (as assessed in Bowler et al., 2014 and the current study) is not reliably impaired, more complex multi-feature (and potentially multi-modal) forms of binding (required for spatial navigation, episodic memory, and episodic future thinking) are in fact impaired. This is something that could be explored in future studies.

The final prediction we made, in terms of between-group performance differences (although not of central interest to the study), was that typically developing children would show greater central coherence, as quantified by the difference in performance between segmented and unsegmented versions of block design. This prediction was broadly confirmed with a marginally significant group difference in the expected direction.

In addition to exploring the basic between-group differences in performance on the main experimental tasks, we also set out to investigate the relations between these tasks and quantitative differences in levels of ASD traits in general, and behavioral inflexibility in particular. The first thing to note, with respect to these analyses, is that results should be interpreted with caution, given the relatively small sample employed. The majority of the predicted, negative correlations failed to emerge (potentially due to limited statistical power). However, within the ASD group a moderate-to-strong and significant negative correlation between scores on the RBS-R (which measures behavioral inflexibility) and navigation performance did emerge. Thus, high levels of inflexible behavior were associated with poorer navigation. Within the comparison group, there was a moderate and significant negative correlation between RBS-R scores and relational memory. However, this latter finding is likely to be a statistical anomaly given that RBS-R scores showed very little variation in this group.

We also explored the relations between each of the tasks that we hypothesized relied on self-projection/scene construction/mental simulation (or some other common cognitive ability such as central coherence or relational memory). Again, very few of the predicted results emerged in the data. Indeed, we observed one result that was completely opposite to what we had expected—within the ASD group, relational memory was moderately-to-strongly and significantly negatively correlated with navigation performance. Notably, the current study failed to replicate our previous finding that spatial navigation was significantly, positively related to episodic memory and ToM in adults with ASD (Lind et al., 2013).

A further surprising finding was that our measures of episodic memory and episodic future thinking were significantly correlated with semantic event knowledge within the ASD group only. Given that both episodic memory and episodic future thinking are thought to rely to some extent on support from the semantic memory system (general event knowledge or “scripts” stored in semantic memory are thought to provide the foundations for episodic retrieval and simulation; e.g., Hudson and Mayhew, 2009; Martin-Ordas et al., 2014), one would have expected to see at least small positive relations within both groups. One possible interpretation is that the current findings reflect an over-reliance on semantic event knowledge among children with ASD, relative to their typically developing counterparts. This may potentially imply that children with ASD were engaging in something more akin to semantic memory based event recall and “semantic future thinking” (Atance and O'Neill, 2005), rather than genuine episodic memory and episodic future thinking (i.e., true “mental time travel”; Wheeler et al., 1997) when completing the past and future events conditions of the task.

In terms of the clinical implications of the current findings, impairments in navigation, episodic memory, and episodic future thinking could potentially have significant consequences for people with ASD. In particular, they suggest that people with the disorder may be more prone to getting lost in their daily lives and have difficulty anticipating future events (which would make planning particularly challenging). With respect to navigation, in particular, it might be possible to develop intervention strategies to improve the skills of individuals with ASD. Relevant to this, we were recently contacted by the mother of a teenage boy with ASD (and accompanying moderate learning disability), who had seen our previous study of navigation among adults with ASD (Lind et al., 2013). She was particularly interested in the findings because they stood in stark contrast to her son's exceptional spatial navigation skills. She described in detail how from an early age he has been able to help the rest of the family find their way around in different environments—urban and rural—and how he always remembers where they have parked the car! She explained that once he has visited a place once, it appears to be imprinted in his memory—he can guide you back to it even when years have elapsed. Perhaps the key to this young man's impressive abilities is that he has a special interest in all things navigation-related (maps, Google Earth etc.) and has developed his own strategies for learning the topographical layout of the places he visits—e.g., finding an elevated position and looking down on his surroundings. This individual case highlights the possible utility of developing intervention strategies that may enhance the navigation skills of individuals with ASD who have difficulties in this domain. This is something that we hope to explore in future research.

In sum, the finding of concurrent impairments in spatial navigation, episodic memory, and episodic future thinking in children with ASD is consistent with our hypothesis that self-projection/scene construction/mental simulation is impaired in ASD (although we acknowledge that other interpretations are possible). Certainly, postulating impairment in one of these putative underlying cognitive processes provides a parsimonious explanation for the results. Difficulties with mentally re-experiencing or pre-experiencing past or future events in the event description task, and with generating a cognitive map of the memory island environment could well be explained by a more fundamental problem with mentally simulating alternative spatial or temporal perspectives (i.e., self-projection) or with generating and maintaining a complex and coherent scene in mind (i.e., scene construction). In other words, children (and adults) with ASD may have difficulties with the representational processes required for mental simulation. We hope that the current study will pave the way for larger-scale, future research to explore the relations between these abilities using a cognitive-experimental approach, rather than merely a neuro-scientific one.

### Conflict of interest statement

Oregon Health & Science University (Jacob Raber's employer) sometimes receives a modest, one-time licensing fee from laboratories that use the Memory Island software (although no payment was received for its use in the present study). The authors declare that the research was conducted in the absence of any commercial or financial relationships that could be construed as a potential conflict of interest.
