# Supplementary material for: Spatial navigation, episodic memory, episodic future thinking, and theory of mind in children with autism spectrum disorder: evidence for impairments in mental simulation?
Source: Front Psychol. 2014 Dec 5;5:1411. doi: 10.3389/fpsyg.2014.01411 (PMC4256988; doi:10.3389/fpsyg.2014.01411)
Supplement: Supplementary file 1 [file DataSheet1.DOCX]

**Appendix 1**

Phrasing of test questions across each condition of the event description (episodic memory/prospection) task

*Past Event Condition*

What did you eat for breakfast this morning?

What did you eat for your evening meal yesterday?

What did you do at bedtime last night?

What food did you get the last time you went food shopping?

What did you do the last time you went to the park?

What did you eat the last time you went to a restaurant?

What did you do the last time you went on a school trip?

What did you do on your last birthday?

What did you do last Christmas day?

*Semantic Event Knowledge Condition*

What do you eat for breakfast?

What do you eat for your evening meal?

What do you do at bedtime?

What food do you get when you go food shopping?

What do you do when you go to the park?

What do you eat when you go to a restaurant?

What do you do when you go on a school trip?

What do you do on your birthday?

What do you do on Christmas day?

*Future Event Condition*

What are you going to eat for breakfast tomorrow?

What are you going to eat for your evening meal tonight?

What are you going to do at bedtime tonight?

What food are you going to get the next time you go food shopping?

What are you going to do the next time you go to the park?

What are you going to eat the next time you go to a restaurant?

What are you going to do the next time you go on a school trip?

What are you going to do on your next birthday?

What are you going to do next Christmas day?
